# PROACTIVE-AI: An Intelligent Dashboard for Assessing and Predicting of FDA-approved AI Software Performance in Real-World Clinical Scenarios

**DOI:** 10.21203/rs.3.rs-10099224/v1

**Published:** 2026-07-22

**Authors:** Isadora Oliveira Grasel, Naveena Gorre, Issam El Naqa

**Affiliations:** 1 H. Lee Moffitt Cancer Center & Research Institute, Department of Machine Learning, Tampa, FL, USA.; 2 University of South Florida, College of Engineering, Tampa, FL, USA.

**Keywords:** Artificial intelligence, FDA devices, real-world applications, recall prediction, benefit-risk assessment

## Abstract

Recent years have witnessed a surge in FDA approved AI tools for healthcare applications. While this growth offers considerable potential benefits for clinical practice, it also introduces substantial challenges related to ethics, regulation, and patient safety. These challenges are further compounded by previously documented gaps in the regulatory approval pathway. These gaps include inconsistent pre-market evaluation practices, over-reliance on retrospective studies, and the limited systematic post-market surveillance of AI devices in real-world clinical settings. Using publicly available FDA data, we developed therefore an interactive web-based dashboard for assessing and predicting the performance of FDA-approved AI software, called PROACTIVE-AI, for the purpose of pro-viding the user with a structured guidance on the anticipated performance of AI-enabled medical devices in real-world clinical settings. The dashboard supports exploratory analysis by diverse stakeholders via knowledge graph visualization and longitudinal trend monitoring of performance indicators, including device recalls and safety-related issues. In addition, PROACTIVE-AI incorporates an AI-aided post-market surveillance risk assessment calculator, derived from historical recall data, to identify device characteristics and con-textual factors associated with elevated deployment risk. Our findings using the PROACTIVE-AI dashboard highlight some of the important challenges related to real-world monitoring and accountability of deployed AI medical devices. Furthermore, it illustrates the potential value of such dashboard in narrowing the trust gap surrounding AI in healthcare by providing quantitative metrics of expected clinical performance and recall-related risk factors.

## 1 Introduction

Recent years have witnessed tremendous growth in AI applications for healthcare in areas spanning computer-aided diagnosis, patient triage, automated retrieval of electronic health records, documentation of doctor-patient encounters, clinical trial matching, drug discovery, and clinical decision support [1]. According to Menlo Ventures’ research in 2025, 22% of healthcare organizations have implemented domain-specific AI tools, a 7x increase over 2024 and 10x over 2023 [2]. This has led to an increase in the number of FDA-cleared AI-enabled medical devices in the United States [3]. This included the FDA release of ELSA (Enterprise Language Support Assistant), a generative AI tool to optimize the agency’s efficiency. However, this growth is not without deployment and translation challenges related to ethical, regulatory, and patient safety considerations [4, 5]. Issues affecting AI medical device regulation and their impact on clinical practice has been the subject of debate in the literature. Wu et al. [6] highlighted several issues related to the lack of consistent pre-market evaluation practices, over-reliance on historical retrospective single-site studies even for high risk scenarios, and the lack of post-market surveillance of AI devices. Abulibdeh et al. [7] further highlight gaps in safety evaluations and ethical considerations and they call for more adaptive, community-engaged regulatory approach that balances innovation with patient safety. These can lead to biases and unintended consequences in clinical practice. These issues are further exacerbated in the context of generative AI technologies [8]. A recent JAMA summit recognized that AI will disrupt every part of health and health care delivery in the coming years, with important health effects. Many of these effects (good or bad) are often not quantified and are likely outside the purview of the regulatory apparatus [9]. This begs the need to develop tools and resources to assess and predict potential benefits and possible risks of AI tools post FDA clearance, as a guide for clinical providers and their medical institutions, which motivated the current work.

The fast-increasing proliferation of Artificial Intelligence-Enabled Medical Devices (AIMD) warrants significant attention. As of July 2025, the U.S. Food and Drug Administration (FDA) has approved more than 1,300 AI/ML-enabled medical devices for market use, and more than 80% of those were cleared in the last five years. However, the agency’s approach to post-market introduction surveillance remains highly passive, relying on the product providers to voluntarily report adverse events and recalls rather than proactively risk tracking [10, 11]. This results in a serious blind spot where little is known about how these devices behave in real-world conditions and even less about what factors may lead to their failure.

Part of this gap stems from a fundamental mislabeling of algorithmic products as devices. The term device implies hardware, a physical tool with fixed form, known properties at approval, and unlikely to change after deployment. Most traditional medical devices, such as a stent, an infusion pump, and a surgical staple, behave in this way, so their operating characteristics do not change after leaving the manufacturing process and reaching healthcare providers and patients. AI/ML products behave the opposite way, being expected to learn from real-world data and adapting to achieve better performance. However, if the goal is for algorithms to be retrained and updated, a model cleared in 2020 for chest X-ray diagnostics could yield substantially different outputs in 2025, for example, not because the “device” itself changed, but because it encountered a distinct patient population, clinical environment, or imaging equipment [12]. The lack of post-market surveillance on current regulatory frameworks does not establish a standardized mechanism for detecting, reporting, or fixing such changes once a device is on the market [13].

The lack of accessibility to recall data adds to this issue. When a device is recalled, three important questions of clinical relevance arise: Who determined that a recall was warranted? On what basis? What kind of failure triggered the recall? [14] And if the device returns to the market, what evidence proves that the underlying problem has been resolved? In practice, most recalls are conducted voluntarily by the manufacturer and, in rare cases, the FDA will order a recall if the manufacturer refuses to act [15]. A healthcare institution’s team evaluating whether to deploy a given AI diagnostic tool has no readily accessible, synthesized record of that device’s safety history [16, 17].

This lack of transparency has direct consequences for patient protection and trust. Patients are rarely informed when an AI/ML system contributes to their diagnosis or treatment recommendation, and legal frameworks governing liability in AI-assisted care remain unsettled. When an algorithmic error contributes to a misdiagnosis, questions of responsibility diffuse across the software developer, the device manufacturer, the deploying institution, and the treating physician, with no consensus on how courts or regulators should enforce accountability. In the absence of clear disclosure obligations, patients cannot meaningfully consent to the role of AI in their care, and clinicians cannot exercise informed judgment about which tools to trust [18].

Together, these gaps — in post-market oversight, in regulatory vocabulary, in data accessibility, and in legal accountability — present a concrete challenge for healthcare institutions. How should a hospital evaluate whether a newly cleared AI/ML device represents a sound clinical investment? What signals, drawn from a device’s regulatory and design history, predict that it is more or less likely to be recalled? And how can that predictive information be made available not only to specialists navigating FDA databases, but to the broader public that depends on these systems?

This paper addresses those questions through an empirical analysis of FDA-cleared AI/ML medical devices. Using a dataset of approximately 1,300 unique devices spanning 1995 to 2025 — compiled from FDA’s AI-Enabled Medical Devices List records — we examine if historical device characteristics can accurately predict the probability that a given device will be recalled and what factors indicate a higher likelihood of recall. Finally, we also discuss how this predictive information can be structured and disseminated so that it is accessible to patients, healthcare providers, and policymakers who lack the technical resources to navigate primary regulatory data sources.

## 2 Material and Methods

**Fig. 1:**
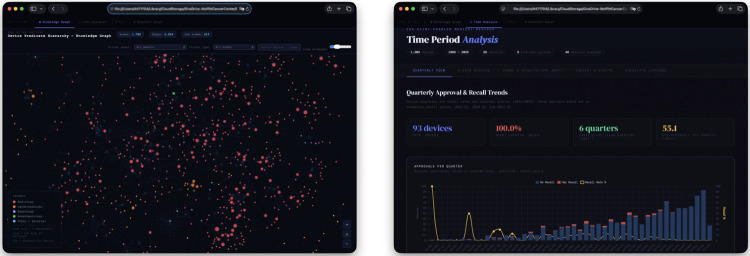
PROACTIVE-AI dashboard for supporting exploratory analysis of FDA AI-based medical devices by the different stakeholders via knowledge graph visualization (a) and longitudinal analysis (b).

Managing and interpreting over 1,300 FDA-approved AIMDs across 30 years presents challenges, especially when drawing inference-based connections among the devices, which static figures cannot adequately address. Therefore, we developed an interactive web-based dashboard called **PROACTIVE-AI: P**redictive **R**eal-world **O**versight, **A**ssessment, **C**ompliance, **T**rack**I**ng, and sur**VE**illance of AI. The PROACTIVE-AI dashboard can support exploratory analysis by clinicians, regulators, and researchers. The dashboard integrates recall records sourced from Lee et al.[14] with predicate relationship data extracted from publicly available FDA decision summary PDFs. The PROACTIVE-AI dashboard is organized into two primary modules — a *Knowledge Graph* and a *Time Analysis* panel, as shown in Figure 1. In addition to an AI-aided post-market surveillance risk assessment calculator, which are discussed in following sections.

### 2.1 Knowledge Graph Analysis

**Fig. 2:**
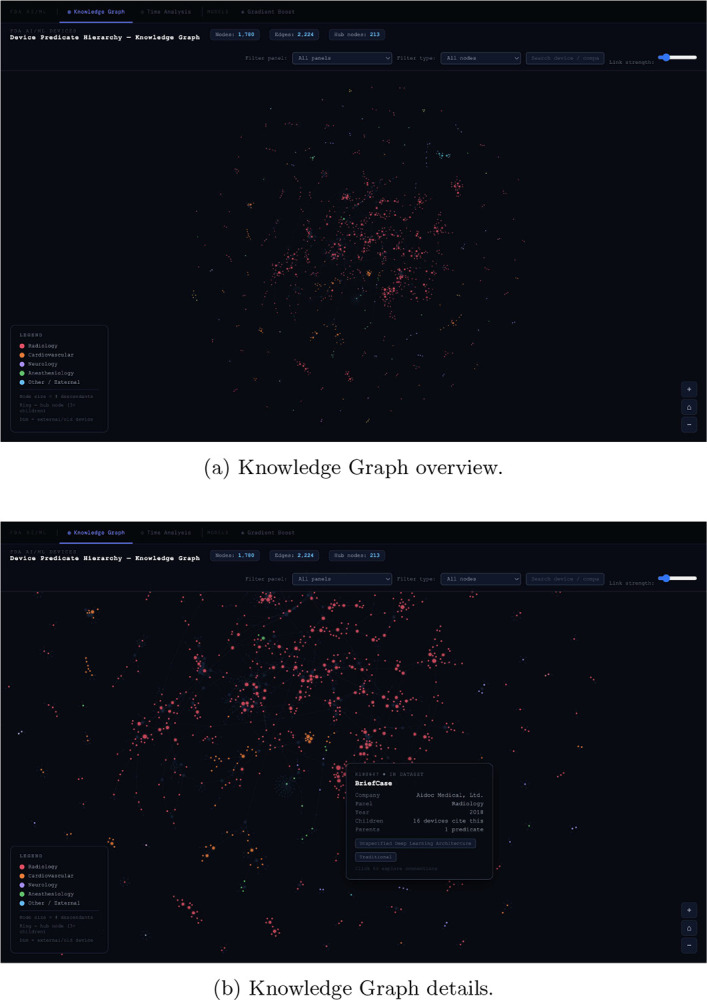
PROACTIVE-AI dashboard: Knowledge Graph panel with an overview (a) and detailed device card information (b).

The *Knowledge Graph* analysis module is based on creating a network of related devices. The reference links are extracted from FDA decision summary PDF files, which were modeled as a directed graph rendered as an interactive force-directed visualization using D3.js, JavaScript library for bespoke data visualization, as shown in Figure 2a. Nodes are color-coded by clinical panel and scaled by number of descendants, with hub nodes — defined as devices with three or more direct children — visually distinguished by a surrounding ring. Users can filter the graph by clinical panel and node type, search for specific devices or companies, and adjust the link strength to explore network density. Hovering over any node displays a detail card with the device name, manufacturer, clearance year, number of citing devices, predicate count, and AI architecture classification, shown in Figure 2b.

### 2.2 Temporal Analysis

**Fig. 3:**
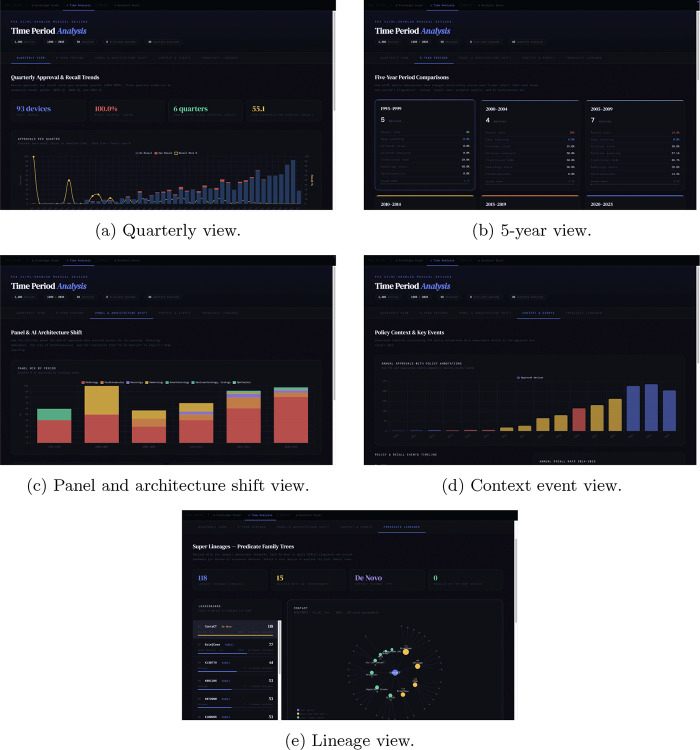
PROACTIVE-AI dashboard: Time Analysis panel with (a) quarterly, (b) 5-year, (c) panel and architecture shift, (d) context event, and (e) lineage views.

The *Time Analysis* module organizes longitudinal patterns from 1995 to 2025 across quarters and five year periods. The *Quarterly view* presents device approvals and recall rates per calendar quarter, distinguishing recalled from non-recalled devices and overlaying recall rate as a continuous trend line, enabling identification of anomalous periods (Figure 3a). The *Five-Year Periods view* summarizes each cohort’s structural fingerprint — recall rate, deep learning adoption, clinical trial prevalence, and panel composition — in comparable cards that make cross-era shifts immediately readable (Figure 3b). The *Panel and Architecture Shift view* tracks how the clinical specialty mix and AI architecture type have evolved across periods (Figure 3c). The *Context and Events view* annotates annual approval volumes with key FDA policy milestones, providing regulatory context for observed shifts in clearance and recall patterns (Figure 3d). Finally, the *Predicate Lineage view* aims to complement this by ranking devices according to the size of their descendant networks, enabling users to explore full family trees and trace how influence propagates across successive clearances (Figure 3e).

### 2.3 PROACTIVE-AI dashboard: Post-market Surveillance Risk Assessment Tool

The PROACTIVE-AI dashboard also includes an AI-based tool that can aid stakeholders (e.g., clinicians, regulators, and researchers) in post-market surveillance analysis of FDA approved devices and predict the associated risk with these approved or possibly will be approved medical devices. This risk prediction is calculated based on previously reported FDA recall data. This AI tool can also help identify factors associated with higher deployment risk. In the following, we describe the steps involved in this tool development, its evaluation, and provide interpretation of its analysis.

#### 2.3.1 FDA Data Pre-processing

The recalled [14] and non-recalled FDA datasets, collected here from publicly available FDA resources, exhibited different structures 82 and 21 columns/features, respectively. This discrepancy was primarily due to the presence of one-hot encoded categorical variables in the recalled dataset. To ensure consistency across datasets, one-hot encoded categorical features in the recalled dataset were reconstructed into their original categorical form. This was achieved by identifying all columns sharing a common prefix and selecting the active category per observation based on the maximum encoded value. To harmonize the feature space, variables present in the non-recalled dataset but absent in the recalled dataset were introduced and assigned a uniform placeholder value (“Unavailable”). Finally, both datasets were aligned to an identical feature schema and concatenated to form a unified dataset for subsequent analysis.

The final feature set consisted of the following variables: Submission Number, Type of Review, Any Clinical Analysis, Formal Clinical Trial, N of Clinical Trial (Patients not images), Device, AI Architecture, Clinical Service, Date Approved, Company, Panel, Product Code, Decision Code, Third Party Review, PDF Summary Available, Predicate Count, and label. Missing values in the combined dataset were imputed using a large language model (GEMINI-3.1-PRO). For each device, structured prompts were used to extract key attributes. The imputed values were then merged back into the dataset using device identifiers, primarily completing missing entries in the recalled class (label = 0) which resulted in 1243 devices for Class 1 (non-recalled) and 59 for Class 0 (recalled). The dataset was subsequently partitioned into training, validation, and test subsets using stratified sampling to preserve class distribution across splits. Subsequently, 20% of the data were withheld as an independent test set, while the remaining 80% were further divided into training and validation subsets.

#### 2.3.2 AI Risk Assessment Model Development

A CatBoost gradient boosting classifier was developed to distinguish recalled devices (label = 0) from non-recalled devices (label = 1). CatBoost is an ensemble machine learning algorithm that builds multiple decision trees sequentially, where each new tree attempts to correct errors made by previous trees [19]. The algorithm was selected for its strong performance on structured tabular data and its ability to natively handle categorical variables without extensive pre-processing. Categorical features were identified automatically and provided directly to the model. To address class imbalance, class weights were applied based on the ratio of non-recalled to recalled samples in the training set. The model was trained using 287 boosting iterations, a learning rate of 0.03, and a tree depth of 6, with Log loss as the objective function and AUC as the evaluation metric. Training was performed using a fixed random seed (random state = 42), and the best-performing iteration on the validation set was retained. To further assess model robustness and generalizability, repeated stratified cross-validation was performed using a 5-fold, 5-repeat Repeated StratifiedKFold strategy. Stratification preserved the class distribution of recalled and non-recalled devices across folds. For each iteration, the CatBoost classifier was retrained using the same hyper parameters with he class-weighting approach described above.

#### 2.3.3 Evaluation metrics

The CatBoost classifier performance was evaluated using Receiver Operating Characteristic Area Under the Curve (ROC-AUC), Precision–Recall Area Under the Curve (PR-AUC), classification reports, and confusion matrix analysis. In addition, Top-K evaluation metrics were used to assess the model’s ability to prioritize high-risk devices, while risk scores were stratified into low, medium, and high-risk categories using the 70th and 90th percentile thresholds of the training score distribution.

#### 2.3.4 AI model Interpretability

To improve transparency and interpretability of the CatBoost model, the SHapley Additive exPlanations (SHAP) was applied [20]. A TreeExplainer was used to compute SHAP values for the trained CatBoost model on the held-out test set. Both global and local interpretability analyses were performed. Global feature importance was derived using the mean absolute SHAP values, providing an overall ranking of feature influence. This allowed evaluation of both the magnitude and direction of feature effects within the model’s decision space.

To explore the underlying structure of the dataset and assess separability between recalled and non-recalled devices, Uniform Manifold Approximation and Projection (UMAP) was applied [21]. The transformed feature space was projected into a two-dimensional embedding using UMAP with 15 nearest neighbors and a minimum distance parameter of 0.1, with a fixed random seed (random state = 42) to ensure reproducibility.

## 3 Results

### 3.1 PROACTIVE-AI Dashboard Visualization

As mentioned earlier, the PROACTIVE-AI dashboard is organized into two primary modules — a *Knowledge Graph* (KG) and a *Time Analysis* (TA) panels. The KG of the 1303 FDA-approved AI devices analyzed was modeled as a directed graph comprising 1,780 nodes and 2,224 edges as seen in Figure 2a, with option tom visualize detailed device information (Figure 2b. The TA module covers longitudinal patterns across five complementary sub-views of the 1,303 devices from 1995–2025, spanning 46 quarters (Figure 3a) and six five-year periods (Figure 3b). An in depth view of trends are captured in the *Panel and Architecture Shift view*, such as the rise of cardiovascular devices and the gradual transition from unspecified to explicit deep learning architectures (Figure 3c). The *Context and Events view* can provide regulatory context for such observed event shifts in clearance and recall patterns of these devices (Figure 3d). These events can be traced back retrospectively using the *Lineage view*, enabling users to conduct fault tree analyses (Figure 3e) of recall events, if they choose so.

### 3.2 AI Risk Assessment Model Performance

To interpret model predictions, probabilistic outputs from the CatBoost classifier were converted into recall risk scores representing the predicted likelihood of device recall. Devices were subsequently stratified into low, medium, and high-risk categories based on percentile thresholds derived from the predicted risk distribution. This stratification enabled identification of devices associated with elevated predicted recall risk and provided a more interpretable framework for downstream risk assessment. The resulting risk distribution demonstrated clear separation between lower risk and higher risk devices, indicating that the model produced well calibrated probabilistic estimates suitable for risk prioritization. These risk scores and categorical risk levels were further integrated into an interactive dashboard framework to support visualization, filtering, and identification of high-risk medical devices for potential regulatory monitoring and decision support.

#### 3.2.1 Internal Cross-validation Performance

To evaluate model robustness and generalizability, repeated stratified cross-validation was performed using a 5-fold, 5-repeat validation strategy. Across all validation folds, the CatBoost classifier achieved a mean ROC-AUC of 0.993 ± 0.014 and a mean PR-AUC of 0.967 ± 0.042. Performance remained consistently high across repeated sampling iterations, with ROC-AUC values ranging from 0.941 to 0.99 and PR-AUC values ranging from 0.854 to 0.99.

**Fig. 4:**
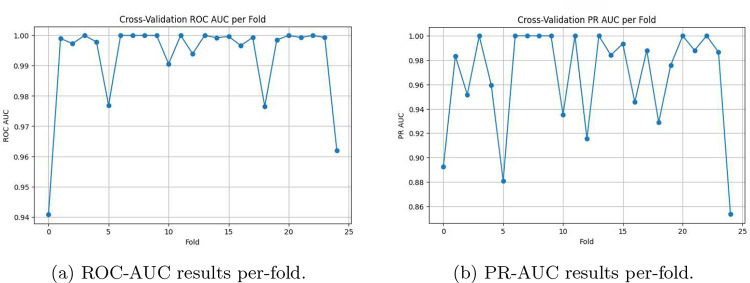
Five-repeat 5-fold cross-validation performance of the CatBoost classifier. The results indicate a relatively stable performance across folds.

These findings demonstrate that the model maintained stable predictive performance across different data partitions and was not strongly dependent on a single train–test split, supporting the robustness and generalizability of the proposed classification framework. The corresponding ROC-AUC and PR-AUC cross-validation results are presented in Figure 4.

#### 3.2.2 Independent Testing Performance

**Fig. 5:**
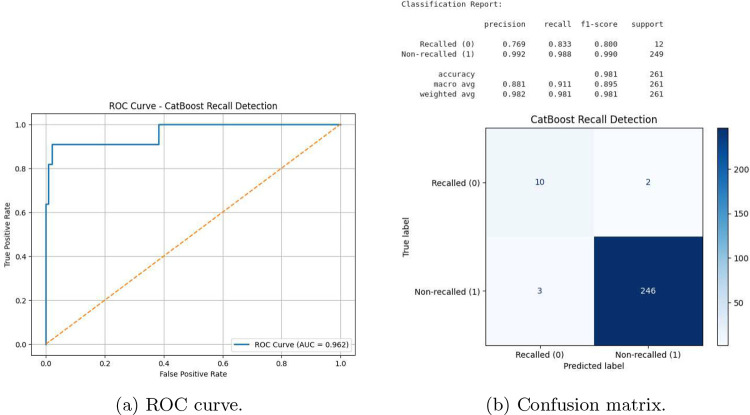
Performance of the CatBoost classifier on the test dataset (n=261). (a) ROC curve, and (b) confusion matrix.

The CatBoost classifier demonstrated strong discriminatory performance in identifying recalled medical devices from non-recalled devices on the independent test dataset (n=261). The model achieved a Receiver Operating Characteristic Area Under the Curve (ROC-AUC) score of 0.961 and a Precision–Recall Area Under the Curve (PR-AUC) score of 0.886. The ROC curve shown in Figure 5a further illustrates the model’s classification capability across varying decision thresholds.

To further evaluate classification behavior, a confusion matrix and classification report were analyzed on the test dataset. The model achieved an overall accuracy of 98.1%, with strong predictive performance across both classes. For recalled devices (Class 0), the model achieved a precision of 0.769, recall of 0.833, and F1-score of 0.800, indicating that the majority of recalled devices were correctly identified despite the substantial class imbalance. For non-recalled devices (Class 1), the classifier achieved precision and recall values of 0.98.

The confusion matrix shown in Figure 5b demonstrates that most recalled and non-recalled devices were correctly classified, with only a small number of false positive and false negative predictions. Importantly, the relatively high recall for the recalled class suggests that the model was effective in identifying high risk devices while maintaining low overall misclassification rates.

#### 3.2.3 Interpretability results

**Fig. 6:**
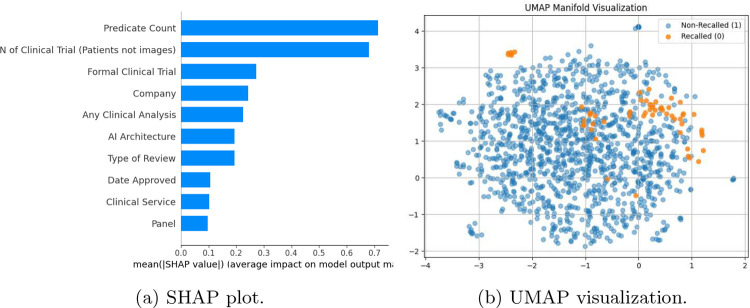
Interpratebility analysis of AI risk assessment module on testing set. (a) Shap plot, and (b) UMAP visualization.

SHAP analysis (Figure 6a) was used to interpret the CatBoost model on the test set and quantify feature contributions to recall prediction. The results identified N of Clinical Trials (Patients not images), Company, and Predicate Count as the top three most influential features based on mean absolute SHAP values. Higher numbers of clinical trials emerged as the most influential feature in the SHAP analysis, contributing strongly to the model’s prediction of recall versus non-recall outcomes. The Company feature also showed strong influence, suggesting manufacturer-specific variation in predicted risk. In addition, the SHAP dependence analysis indicated that lower values of Predicate Count were more strongly associated with recall predictions, whereas higher values generally contributed toward non-recall predictions. Overall, SHAP analysis demonstrated that model predictions were primarily driven by a subset of clinically and regulatory relevant features.

Building on these findings, UMAP (Figure 6b)was used to visualize the global structure of the dataset in a reduced dimensional space. The projection revealed strong separation between recalled and non-recalled samples forming distinct class clusters and only limited overlap further supporting the observed discriminatory results of the AI risk assessment module.

## 4 Discussion

This work demonstrated a new interactive web-based dashboard for assessing and predicting the performance of FDA-approved AI software (PROACTIVE-AI). It builds directly on the dataset compiled by Lee et al. [14] in their cross-sectional study of FDA-approved AIMD recalls, which identified lack of clinical validation and publicly traded manufacturer status as the two strongest independent predictors of recall. While Lee et al. provided the first systematic characterization of recall epidemiology in this device class, their analysis was descriptive. The PROACTIVE-AI dashboard presented here extends that foundation toward prediction: rather than characterizing which device populations carry elevated risk retrospectively, the CatBoost classifier assigns prospective recall risk scores to individual devices at the time of clearance, enabling earlier and more targeted post-market surveillance. The convergence between their findings and our model’s SHAP-derived feature importance is notable. Lee et al. [14] reported that devices without reported clinical validation had significantly more recalls per device and were associated with larger-scale recalls than those with retrospective or prospective validation. In our model, the number of clinical trial patients emerged as the single most influential feature in the SHAP analysis, contributing more strongly to recall prediction than any other variable. This alignment across two independent methodological approaches strengthens the case that clinical validation depth is not merely correlated with recall risk but is a robust signal detectable from pre-clearance submission data alone.

The Predicate Count feature offers a complementary finding not captured in Lee et al.’s framework [14]. SHAP dependence analysis showed that lower predicate counts were more strongly associated with recall predictions, while higher counts contributed toward non-recall predictions. One interpretation is that devices with fewer established predicates represent more novel algorithmic approaches, grounded on the basis of thinner substantial equivalence claims. This aligns with broader concerns in the literature about the 510(k) pathway’s suitability for AI/ML products, unlike traditional hardware devices, whose performance properties are fixed at manufacture, AI/ML device behavior can diverge substantially from that of their predicates once deployed into real-world clinical environments [22]. Lee et al. also found that 43.4% of recalls occurred within the first 12 months of clearance, approximately double the rate reported for all 510(k) devices, a finding consistent with a deployment gap between regulatory approval and real-world clinical performance. The temporal concentration of early recalls provides additional motivation for the risk stratification framework described here: a tool that flags high-risk devices at clearance could trigger enhanced surveillance precisely during the window when failures are most likely to emerge.

There are important differences in scope worth acknowledging. Lee et al. studied 950 AIMDs matched to recall entries as of November 2024, whereas the dataset used in the present analysis spans a broader time window and includes devices from both recalled and non-recalled populations, resulting in a different device count after de-duplication. Additionally, their multi-variable model adjusted for manufacturer commercialization type — a variable not included in our feature set — which was associated with an odds ratio of 5.9 in their analysis. Future iterations of the predictive pipeline could incorporate company type as a feature, suggesting that adding it could improve classifier performance further. Combined, the findings support a layered view of AIMD recall risk: structural factors identifiable at clearance — limited clinical evidence, novel predicate chains, and manufacturer type — are predictive of post-market failure, and a machine learning framework can operationalize these signals into actionable risk scores for regulators, health systems, and clinical decision-makers, as recommended by the JAMA AI summit [9].

## 5 Conclusions

We have developed and demonstrated PROACTIVE-AI, a friendly interactive web-based dashboard for assessing and predicting the performance of FDA-approved AI software in real-world clinical settings. The PROACTIVE-AI dashboard supports exploratory network and longitudinal analyses that enable capturing trends in reported performance including recalls and safety-related issues. It also provides an AI-aided post-market surveillance risk assessment calculator that can identify factors associated with higher deployment risk. Our preliminary analysis confirmed quantitatively the observation that devices without reported clinical validation had significantly more recalls per device. It also identified that lower predicate counts were more strongly associated with recall risk.

## Data Availability

The datasets generated and/or analyzed during the current study are not publicly available as they consist of a derived dataset constructed from publicly available FDA data through a multi-stage data processing, wrangling, and feature engineering pipeline. The underlying FDA data are publicly accessible (https://www.fda.gov/medical-devices/software-medical-device-samd/artificial-intelligence-enabled-medical-devices). The recall event dataset is credited to Lee et al. [14]. The final curated and pre-processed dataset for predictive model development is available from the authors upon reasonable request.

## Code Availability

The underlying code for this study is available through the GitHub repository: https://github.com/isadoragrasel/fda-dashboard, which will be made public upon the manuscript publication.
